# Relationship between social network and individual performance of core members from aged care services social organizations: cross-sectional study

**DOI:** 10.1186/s12877-023-03837-x

**Published:** 2023-02-23

**Authors:** Zhengsheng Wang, Xuefei Chen, Kai Ji, Lingzhi Sang, Zhongliang Bai, Ren Chen

**Affiliations:** 1 School of Health Services Management, Anhui Medical University, Hefei, 230032 China; 2 Faculty of Education, Health and Wellbeing, University of Wolverhampton, Wolverhampton, WV1 1QU UK; 3 Suzhou Hospital Affiliated to Anhui Medical University, Suzhou, 234000 Anhui China

**Keywords:** Social network, Aged care services, Individual performance, Social organizations, Core members

## Abstract

**Background:**

The social network of core members can affect the performance of the organization, while there is a lack of research on the relationship between the social network of core members of social organizations and individual performance in the field of aged care services. This study aimed to explore the relationship between social network and individual performance of core members from social organizations engaged in aged care services and explore measures to promote the development of aged care services.

**Methods:**

We used a multi-stage stratified sampling method to conduct a cross-sectional study and collected the required data in six cities in Anhui Province, China. Univariate analysis and binary logistic regression were used to estimate the relationship between social network and individual performance.

**Results:**

Our results indicated that core members with higher social network scores were more likely to yield better individual performance, including receiving awards or recognitions related to aged care services (AOR=2.534; 95% CI: 1.397-4.596). Moreover, teams led by the core members were more likely to receive awards or recognitions related to aged care services (AOR=2.930; 95% CI: 1.740-4.933). The core members or the teams led by them were more likely to be reported by the media (AOR=1.748; 95% CI: 1.030-2.966) and participate in the drafting or discussion of local aged care service standards or service specifications (AOR=2.088; 95% CI: 1.093-3.911). In addition, demographic variables such as gender, marital status, and education of core members were significantly related to their performance (*P*<0.05).

**Conclusions:**

The social network of core members of aged care service social organizations has an impact on their individual performance. To improve the performance of the core members of senior citizens services and organizations, relevant measures should be taken from the government, social organizations and core members to strengthen the social network construction of core members.

**Supplementary Information:**

The online version contains supplementary material available at 10.1186/s12877-023-03837-x.

## Background

Since the 21^st^ century, population aging has become a global phenomenon, critically impacting human health and health care planning [1]. As a result, aged care services face enormous pressures, especially in China [2]. Therefore, it is necessary to seek social forces to participate in aged care services. The cross-sectoral cooperation theory states that the cooperation between government and social organizations can be helpful in the implementation of routine work [3]. The participation of social organizations in different fields of society, including health care for older people, has been found to play an extremely important role [4–6]. In this regard, the contribution of social organizations to public health, especially in disease prevention and health promotion, has been well recognized [7, 8]. Accordingly, the analysis of social organizations that care for the aged can help develop new measures to actively respond to population aging.

Social network refers to a social phenomenon composed of entities connected by various relationships, reflecting the interaction and interconnection between people [9]. Many studies have focused on the relationship between social network and health in recent years, especially in the aged [10, 11]. Nonetheless, the impact of an individual's social network on organizational member performance remains largely understudied. The term "core members" generally refers to founders, leaders, legal representatives, and managers familiar with the organization. As the organization's leaders, they can represent the organization externally and play a very important role in the development of the organization. In a sense, the social network of core members can determine the organization's social network and then influence its development. A study has shown that social network at the workplace can affect the employees' work performance [12], while another study showed that the social network of organizational leaders could affect performance levels, which can, in turn, affect the sustainable development of the organization [13]. Moreover, current evidence suggests that social network has a certain moderating effect on organizational performance [14]. To sum up, it is necessary to explore the relationship between the social network of core members of social organizations in aged care services and individual performance, which will provide the basis for formulating policies to improve aged care.

## Methods

### Study design and data collection

This survey was approved by the Biomedical Ethics Committee of Anhui Medical University (No. 20180181) and was conducted in Anhui Province, China, from November to December 2019.

We used a multi-stage stratified sampling method to collect samples in this study. According to the 2016 statistical data of Anhui Provincial Bureau of Statistics and related research [15, 16], the economic level of Anhui Province is high in the middle, medium in the south, and low in the north. To ensure the representativeness of the study area, we selected Lu'an and Huainan in central Anhui, Anqing, and Chizhou in southern Anhui, and Fuyang and Suzhou in northern Anhui as the survey cities, based on geographical location and economic development level. All districts of each city were taken as the survey area, with a total of 15 districts. Then, we randomly selected half of the social organizations in the aged service field in each district as the survey organizations. Finally, If the number of core members in an organization is less than or equal to 3, all core members in the organization will be included in the survey. If the number of core members in an organization is greater than 3, then 3 of them will be randomly selected for the survey. The core members included the organization founders, organization leaders, legal representatives, and organization managers.

The investigative work was coordinated by the civil affairs departments of each city and district, and the investigators were trained and experienced graduate students from Anhui Medical University. The investigators were divided into 4 teams, and under the leadership of the workers of the civil affairs department, they visited the social organizations one by one and conducted a face-to-face interview questionnaire survey on the subjects. In this study, a total of 315 core members of social organizations involved in aged care services participated in the questionnaire survey, of which 308 (97.8%) core members met the analysis conditions.

### Measure

On the basis of some previous studies and some mature scales, we developed a questionnaire on the social network and individual performance of core members of social organizations in the field of aged care services. After several rounds of consultation with authoritative experts in the professional field, we conducted a preliminary survey in Hefei. Then, the final questionnaire was determined after modification and improvement. The detailed questionnaire can be found in the supplementary file (1).

### Measurement of social network

Based on the World Bank's Social Capital Assessment Tool and previous researches [17, 18], we developed an individual social network measurement questionnaire. The individual social network measurement questionnaire consisted of two parts (social network with government departments and social institutions), with a total of 7 items. The social network with government departments measured the number of acquaintances of core members in the Civil Affairs Department, the number of acquaintances of core members in the Health Committee, the number of acquaintances of core members in the Healthcare Security Administration, and the number of acquaintances in other government departments. And the social network with social institutions measured the number of acquaintances of core members in the Neighborhood Committee, the number of acquaintances in the Federation of Social Organizations as well as the number of acquaintances in other aged care service social organizations. For each item of social network, we used the five-point scale of Likert's to measure the number of acquaintances of the core members (1 = "0", 2 = "1-5", 3 = "6-10", 4 = "11-15", and 5 = "16 and above"). The total score of social network of each survey was obtained by adding the scores for the 7 items, ranging from 7 to 35 points. The Cronbach's α of the social network was 0.885.

### Measurement of individual performance

Based on previous research, we set up the measurement indicators of individual performance of core members of social organizations in the field of aged care services [19]. In this study, five indicators (namely Achievements 1-5) were used to evaluate individual performance after several consultations with authoritative experts in the professional field. The indicators were assessed by the external impact of care services provided by core members, which were objective and could evaluate the achievements of core members in the field of aged care services to a certain extent. The Cronbach's α of the individual performance was 0.623, which achieved the minimum acceptable limit of 0.6 [20, 21]. Achievement 1 represented "Received awards or recognitions related to aged care services". Achievement 2 represented "The team you lead received awards or recognitions related to aged care services". Achievement 3 represents "The media have reported you or the team you lead for the aged care services you were engaged in". Achievement 4 represented "You were a member of an industry associated with the field of aged care services". Finally, Achievement 5 represented "You participated in drafting or discussion of local aged care service standards or service specifications". The achievement was attributed to the core member if the answer was yes. These indicators reflected the influence of core members on aged care services.

### Measurement of other Variables

Data collected on the demographic variables included gender (male, female), age (≤40, 41-49, ≥50 years), ethnicity (Han, others), level of education (junior high school and below, senior high school, college degree and above), marital status (married, others), and holder of a professional title (yes, no). Other information collected included the organization's length of service (≤1year, 2-5years, ≥6years), length of service at the aged care service (≤1year, 2-5years, ≥6years), holder of a practicing certificate (yes, no), holder of a professional qualification certificate (yes, no), prior management training (yes, no) and prior technical business training (yes, no).

### Statistical analysis

First, we tested the normality of the scores associated with social network. For non-parametric variables, the median (interquartile range) was used to express the data and the Chi-square test to examine the differences in individual performance between different demographic groups. Categorical variables were expressed using numbers and percentages. Then, the Mann-Whitney test was used to compare the differences in social network scores between the achievement and non-achievement groups. Finally, we divided the social network scores into high and low categories based on the median score [22]. Taking "individual achievement" as the dependent variable (1 = yes, 0 = no), variables that were statistically significant in the Chi-square test and Mann Whitney test were included in the binary logistic regression model, using the "Enter" method for analysis. The binary regression logistic analysis results were expressed using adjusted odds ratio (AOR) and the related 95% confidence interval (95% CI). All statistical analyses were performed using IBM SPSS Statistics 23.0 software. A P-value < 0.05 was statistically significant.

## Results

### Results of descriptive analysis

The social-demographic characteristics of the included core members of social organizations are shown in Table 1. Among the respondents, 178 were men (57.8%), and 130 were female (42.2%). 56.5% of the respondents were over 50 years old, 92.2% were married, and Han ethnicity accounted for the overwhelming majority (99%). 34.1% of the respondents had a college degree or above, while 79.9% did not have professional titles. 38.6% of the respondents worked in the organization for 2-5 years, and 39.6% worked in aged care services for more than six years. 84.4% of the respondents were not holders of a practicing certificate, while 65.9% obtained a professional qualification certificate. In terms of training experience, 70.5% of the respondents received management training, and 68.2% received technical business training.

**Table 1:** Social demographic characteristics of core members (*N*=308)

Variables	N (%)
**Gender**	
Male	178 (57.8)
Female	130 (42.2)
**Age (years)**	
≤40	74 (24)
41-49	60 (19.5)
≥50	174 (56.5)
**Ethnicity**	
Han	305 (99)
Others	3 (1)
**Education**	
Junior high school and below	91 (29.5)
Senior high school	112 (36.4)
College degree and above	105 (34.1)
**Marital status**	
Married	284 (92.2)
Others	24 (7.8)
**Professional title holder**	
Yes	62 (20.1)
No	246 (79.9)
**Organization's length of service (years)**	
≤1	98 (31.8)
2-5	119 (38.6)
≥6	91 (29.5)
**Length of service in the aged care field (years)**	
≤1	77 (25)
2-5	109 (35.4)
≥6	122(39.6)
**Holder of a practicing certificate**	
Yes	48(15.6)
No	260(84.4)
**Holder of a professional qualification certificate**	
Yes	203(65.9)
No	105(34.1)
**Received management training**	
Yes	217(70.5)
sNo	91(29.5)
**Received technical business training**	
Yes	210(68.2)
No	98(31.8)

### Results of univariate analysis

#### Univariate analysis of demographic factors with individual performance

The results of the univariate analysis of the relationship between demographic factors and individual performance are as follows.

For Achievement 1, the influencing factors were "marital status (*P*<0.05)", "organization's length of service (*P*<0.05)", "length of service in the aged care field (*P*<0.001)", "holder of a practicing certificate (*P*<0.05)", "holder of a professional qualification certificate (*P*<0.05)", "prior management training (*P*<0.001)" and "prior technical business training (*P*<0.001)".

For Achievement 2, the influencing factors were "gender (*P*<0.05)", "ethnicity (*P*<0.05)", "organization's length of service (*P*<0.001)", "length of service in the aged care field (*P*<0.001)", "prior management training (*P*<0.001)", and "prior technical business training (*P*<0.05)".

For Achievement 3, the influencing factors were "gender (*P*<0.05)", "age (*P*<0.05)", "level of education (*P*<0.001)", "holder of a professional title (*P*<0.05)", "organization's length of service (*P*<0.05)", "length of service in the aged care field (*P*<0.05)", "holder of a professional qualification certificate (*P*<0.05)", "prior management training (*P*<0.05)" and "prior technical business training (*P*<0.05)".

For Achievement 4, the influencing factors were "gender (*P*<0.05)", "holder of a professional title (*P*<0.001)", "organization's length of service (*P*<0.05)", "length of service in the aged care field (*P*<0.05)", "holder of a practicing certificate (*P*<0.001)", "holder of a professional qualification certificate (*P*<0.05)", "prior management training (*P*<0.05)" and "prior technical business training (*P*<0.05)".

For Achievement 5, the influencing factors were "level of education (*P*<0.05)", "holder of a practicing certificate (*P*<0.05)", "holder of a professional qualification certificate (*P*<0.05)" and "prior management training (*P*<0.05)".

#### Univariate analysis of social networks scores and individual performance

The results of univariate analysis of social network scores and individual performance are shown in Table 2. We found that social network scores could influence achievement 1 (*P*<0.001), achievement 2 (*P*<0.001), achievement 3 (*P*<0.001), and achievement 5 (*P*<0.05).

**Table 2:** Univariate analysis results of social network scores and individual performance (*N*=308)

	Achievement 1		Achievement 2		Achievement 3		Achievement 4		Achievement 5	
	Yes	No	Yes	No	Yes	No	Yes	No	Yes	No
**Social network score(M (IQR))**	17(10.75)	12(7)	16(10)	12(6)	15(9)	12 (8)	15(7.75)	13 (8)	16(10)	13 (8)
***Z***	-4.788		-4.442		-3.934		-1.885		-3.339	
***P***	**<0.001**		**<0.001**		**<0.001**		0.059		**0.001**	

### Results of binary logistic regression analysis

The results of binary logistic regression analysis of the relationship between social networks score and individual performance of core members are shown in Table 3. Only statistically significant variables are shown in Table 3. In the Hosmer-Lemeshow test, the *P*-value of achievement 1, 2, 3, 4, and 5 were 0.609, 0.847, 0.993, 0.252, and 0.633, which were all higher than 0.05, suggesting no significant difference between the predicted value and the actual value, and good fitting of the model.

**Table 3:** Binary logistic regression analysis of social network score and individual performance

Performance	Variables		***P***	AOR (95%CI)
**Achievement 1**	**Marital status**	Others (REF.)		
		Married	0.029	5.728 (1.199-27.356)
	**Length of service in the aged care field (years)**	≤1 (REF.)		
		2-5	0.050	3.801 (0.999-14.465)
		≥6	0.002	8.904(2.277-34.822)
	**Prior management training**	No (REF.)		
		Yes	0.006	3.172(1.388-7.248)
	**Prior technical business training**	No (REF.)		
		Yes	0.035	2.285(1.062-4.918)
	**Social network score**	Low (REF.)		
		High	0.002	2.534(1.397-4.596)
**Achievement 2**	**Gender**	Female (REF.)		
		Male	0.020	1.881(1.105-3.204)
	**Prior management training**	No (REF.)		
		Yes	<0.001	3.239(1.693-6.196)
	**Social network score**	Low (REF.)		
		High	<0.001	2.930(1.740-4.933)
**Achievement 3**	**Level of Education**	Junior high school and below (REF.)		
		Senior high school	0.004	2.561(1.359-4.825)
	**Organization's length of service (years)**	≤1 (REF.)		
		≥6	0.033	3.518(1.109-11.159)
	**Social network score**	Low (REF.)		
		High	0.039	1.748(1.030-2.966)
**Achievement 4**	**Holder of a Professional title**	No (REF.)		
		Yes	0.016	2.497(1.186-5.258)
	**Length of service in the aged care field (years)**	≤1 (REF.)		
		2-5	0.038	4.369(1.087-17.568)
	**Prior management training**	No (REF.)		
		Yes	0.029	2.580(1.104-6.033)
**Achievement 5**	**Social network score**	Low (REF.)		
		High	0.026	2.088(1.093-3.911)

From the results, we found that core members with high social network score were more likely to accomplish achievement 1(*P*<0.05), achievement 2 (*P*<0.001), achievement 3 (*P*<0.05) and achievement 5 (*P*<0.05) than core members with low social network score. gender, Moreover, we found that gender, marital status, and education of core members are also the influencing factors of core members' individual performance (*P*<0.05).

## Discussion

This survey explored the relationship between the social network phenomenon and the individual performance of core members of social organizations in the field of aged care services in Anhui, China. A previous study has shown that people with the richer social network could frequently benefit from this and had greater access to external resources and information [23]. The present study results showed that the social network level of core members had an impact on their performance, as subjects with high social network scores were more inclined to receive awards or recognitions related to aged care services. Moreover, the teams they led were more likely to receive awards or recognitions related to aged care services; the core members or the teams led by them were more likely to be reported by the media for the aged care services they were engaged in and were more likely to participate in the drafting or discussion of local aged care service standards or service specifications. This finding demonstrates that social networking can indeed affect the individual performance of core members, consistent with the literature [24]. The social network of core members can determine the social network of the entire organization. Moreover, core members can establish close contact with other organizations through social network and then acquire knowledge related to work problems, conducive to better individual performance [25]. Indeed, core members with high individual performance are essential to promote the organization's development.

In addition to social network, demographic variables also exerted varying degrees of impact on the individual performance of core members. During the binary logistic regression analysis, our results found that prior management training was the most significant factor affecting the performance of core members besides social network. In China, many managers and front-line staff in nursing homes received no training in aged care, as employees often tend to exhibit lower expertise levels than their counterparts in developed countries. Accordingly, improving aged care services in China faces many difficulties [26, 27]. A relationship has been defined between management training and performance. An increasing body of evidence suggests that prior management training can influence individual performance and organizational performance and promote the achievement of organizational goals [28, 29]. Employees can indeed acquire more knowledge through management training. It has been reported that employees who acquire knowledge in many ways tend to grow faster in the organization [30]. It is well-established that knowledge has a positive effect on individual performance. Through learning, employees can transform their potential into organizational creativity, thereby improving organizational performance [31]. Accordingly, encouraging core members to participate in management-related training may be a good idea to improve the performance of core members. Other variables such as length of service and level of education are also influencing factors for some of the above performance indicators.

Herein, we explored the impact of social network on the individual performance of core members and provided a new perspective for studying the development of social organizations in the field of aged care services.

It should be pointed out that several limitations were present in this study. First of all, given that this is a cross-sectional study conducted in Anhui, it is difficult to determine the causal relationship between social network and individual performance. The research conclusions are thus only applicable to the Anhui Province. Moreover, we used a small sample size, which may be a source of bias.

## Conclusions

Our study found that the social network of core members of social organizations in aged care services in Anhui Province has a certain impact on individual performance. Core members with higher social network level are more likely to make achievements in some aspects, such as receiving awards or recognitions related to aged care services, winning awards or recognitions related to aged care services for their teams, being reported by the media for engaging in aged care services, and being invited to participate in the drafting or discussion of aged care service standards or service specifications. These individual performances of core members can help the organization obtain more resources for development. In order to improve the individual performance of core members and promote the sustainable development of social organizations in the field of aged care services, we put forward suggestions from the following aspects to improve the social network level of core members. First, the government should build a communication network platform for core members of social organizations. Second, social organizations should encourage core members to participate in aged care service industry conferences and forums. Finally, core members should also strengthen contact with other members of social organizations for the aged, and actively expand their own interpersonal network. In addition, both governments and social organizations should strengthen the training of aged care services for core members. This study can provide the basis for future studies and promote new strategies to improve current aged care services.

## Supplementary Information

**Additional file 1.**

## Data Availability

The datasets analysed during the current study are available from the corresponding author on reasonable request.

## Abbreviations

AOR: Adjusted odds ratio
95% CI: 95%: confidence interval
REF: Reference
