# Supplementary material for: Relationship between social network and individual performance of core members from aged care services social organizations: cross-sectional study
Source: BMC Geriatr. 2023 Feb 23;23:108. doi: 10.1186/s12877-023-03837-x (PMC9948443; doi:10.1186/s12877-023-03837-x)
Supplement: Supplementary file 1 — Additional file 1. [file 12877_2023_3837_MOESM1_ESM.docx]

**Questionnaire for core members of social organizations in the field of aged care services**

**Part A Basic information of** **the core members**

| **A** **Basic information** |
| --- |
| **A1 Gender:** ①Male；②Female |
| **A2 Age：** |
| **A3 Ethnicity:** ①Han；②Others |
| **A4 Education：**①Junior high school and below ②Senior high school ③College degree and above |
| **A5 Marital status:** ①Married；②Others |
| **A6 Professional title:** ①Have；②Not have |
| **A7** **Length of service in the organization:** ①≤1year; ②2-5 years; ③≥6 years |
| **A8 Length of service in the aged care field:** ①≤1year; ②2-5 years; ③≥6 years |
| **A9 Have you obtained the practice certificate?** ①Yes；②No |
| **A10 Have you obtained a professional qualification certificate?** ①Yes；②No |
| **A11 Have you received management training related to aged care services?** ①Yes；②No |
| **A12 Have you received technical training related to aged care services?** ①Yes；②No |

**Part B Social network of the core members**

| **Entries:** | ①0 | ②1-5 | ③6-10 | ④11-15 | ⑤16 and above |
| --- | --- | --- | --- | --- | --- |
| **B1.1** Number of your acquaintances in the Civil Affairs Department. |  |  |  |  |  |
| **B1.2** Number of your acquaintances in the Health Committee. |  |  |  |  |  |
| **B1.3** Number of your acquaintances in the Healthcare Security Administration. |  |  |  |  |  |
| **B1.4** Number of your acquaintances in other government departments. |  |  |  |  |  |
| **B1.5** Number of your acquaintances in the Neighborhood Committee. |  |  |  |  |  |
| **B1.6** Number of your acquaintances in the Federation of Social Organizations. |  |  |  |  |  |
| **B1.7** Number of your acquaintances in other aged care service social organizations. |  |  |  |  |  |

**Part C Individual Performance of the core members**

| **C1.1** Received awards or recognitions related to aged care services. | ①Yes | ②No |
| --- | --- | --- |
| **C1.2** The team you lead received awards or recognitions related to aged care services. | ①Yes | ②No |
| **C1.3** The media have reported you or the team you lead for the aged care services you were engaged in. | ①Yes | ②No |
| **C1.4** You were a member of an industry associated with the field of aged care services. | ①Yes | ②No |
| **C1.5** You participated in drafting or discussion of local aged care service standards or service specifications. | ①Yes | ②No |

Investigator：

Quality controller：

Investigation time：
